# Molecular, morphological and survival analysis of 177 resected pancreatic ductal adenocarcinomas (PDACs): Identification of prognostic subtypes

**DOI:** 10.1038/srep41064

**Published:** 2017-02-01

**Authors:** Anna Melissa Schlitter, Angela Segler, Katja Steiger, Christoph W. Michalski, Carsten Jäger, Björn Konukiewitz, Nicole Pfarr, Volker Endris, Markus Bettstetter, Bo Kong, Ivonne Regel, Jörg Kleeff, Günter Klöppel, Irene Esposito

**Affiliations:** 1 Institute of Pathology, Technische Universität München, Munich, Germany; 2 Department of Surgery, University Hospital Heidelberg, Germany; 3 Department of Surgery, Klinikum rechts der Isar, Technische Universität München, Munich, Germany; 4 Institute of Pathology, University Hospital of Heidelberg, Heidelberg, Germany; 5 Molecular Pathology South-Bavaria, Munich, Germany; 6 Institute of Pathology, Heinrich-Heine-University, Düsseldorf, Germany; 7 The Royal Liverpool and Broadgreen University Hospitals, Prescot Street, Liverpool L7 8XP, United Kingdom; 8 Department of General-, Visceral- and Pediatric Surgery, University Hospital Düsseldorf, Heinrich Heine University Düsseldorf, Germany

## Abstract

Pancreatic ductal adenocarcinoma (PDAC) has generally a poor prognosis, but recent data suggest that there are molecular subtypes differing in clinical outcome. This study examines the association between histopathologic heterogeneity, genetic profile, and survival. Tumor histology from 177 resected PDAC patients with follow-up data was subclassified according to predominant growth pattern, and four key genes were analyzed. PDACs were classified as conventional (51%), combined with a predominant component (41%), variants and special carcinomas (8%). Patients with combined PDACs and a dominant cribriform component survived longer than patients with conventional or other combined PDACs. Genetic alterations in at least two out of four genes were found in 95% of the patients (*KRAS* 93%, *TP53* 79%, *CDKN2A/p16* 75%, *SMAD4* 37%). Patients with less than four mutations survived significantly longer (p = 0.04) than those with alterations in all four genes. Patients with either wildtype *KRAS* or *CDKN2A/p16* lived significantly longer than those with alterations in these genes (p = 0.018 and p = 0.006, respectively). Our data suggest that the number of altered genes, the mutational status of *KRAS* and certain morphological subtypes correlate with the outcome of patients with PDAC. Future pathology reporting of PDAC should therefore include the *KRAS* status and a detailed morphological description.

Pancreatic ductal adenocarcinoma (PDAC) is an aggressive tumor with dismal prognosis. The overall 5-year survival rate is only 6% and after curative surgery less than 25%^1^, making PDAC one of the most lethal tumors among solid malignancies^2^. This poor outcome is related to multiple factors, including resistance to chemotherapy and the relatively late stage of diagnosis due to unspecific symptoms and aggressive tumor biology^1^.

Over the last decade major improvements have been made in understanding the mechanisms of molecular carcinogenesis in PDACs^3^,^4^,^5^,^6^,^7^. The first milestone was the discovery of the molecular fingerprint of PDAC that included the common mutations in *KRAS, SMAD4, TP53* and *CDKN2A/p16*^8^. Recent advances in gene sequencing^9^ by introduction of high-throughput molecular methods allowed to further address the genetic complexity of PDAC^10^. The first global analysis of 24 advanced PDACs using comprehensive exome sequencing revealed a high mutation rate with an average of 63 mutations per case connected to 12 core signaling pathways^10^. In 2011, Collisson *et al*. showed that PDACs and murine PDAC cell lines may be stratified by their transcriptional profiles into subtypes with different clinical outcome and drug response^11^. The stratification of these subtypes was associated with three patterns that were termed classical, quasi-mesenchymal and exocrine-like. The terminology of this subdivision, however, is hardly understandable regarding its morphological meaning. In other recent studies, it was reported that intact *KRAS* and the number of mutations in key genes may have prognostic value in PDAC patients^12^,^13^,^14^,^15^,^16^. However, it is unclear so far, whether there are genotype-phenotype correlations that are based on the identification of PDACs with special growth patterns or morphological variants with better survival than classical PDACs, beyond the established pathological parameters of staging and grading^17^.

This study that only involved PDAC patients in whom surgical treatment could be performed, focuses on detailed histological investigation and molecular examination of the mutational status of *KRAS, CDKN2A/p16, TP53* and *SMAD4* in correlation with survival and accurate morphological subtyping. The presented findings show that, according to the analyzed parameters, prognostic relevant subtypes of PDAC can be identified.

## Results

The patients’ clinicopathologic features are summarized in Table 1. Female (45.8%) and male (54.2%) patients were equally represented with a median age at diagnosis of 68 years. Most patients presented with advanced stage of the disease (≥UICC 2b: 73.4%, 130/177). A minority of patients (9.4%) received neoadjuvant treatment (details see Supplementary Table 1). All patients were judged resectable and underwent major pancreatic surgery: pylorus-preserving partial pancreaticoduodenectomy (ppWhipple) 55.4% (98/177), partial pancreaticoduodenectomy (Whipple, classic) 9% (16/177), distal pancreatectomy 18.6% (33/177) and total pancreatectomy 17% (30/177).

### Histological features

**Figure 1:**
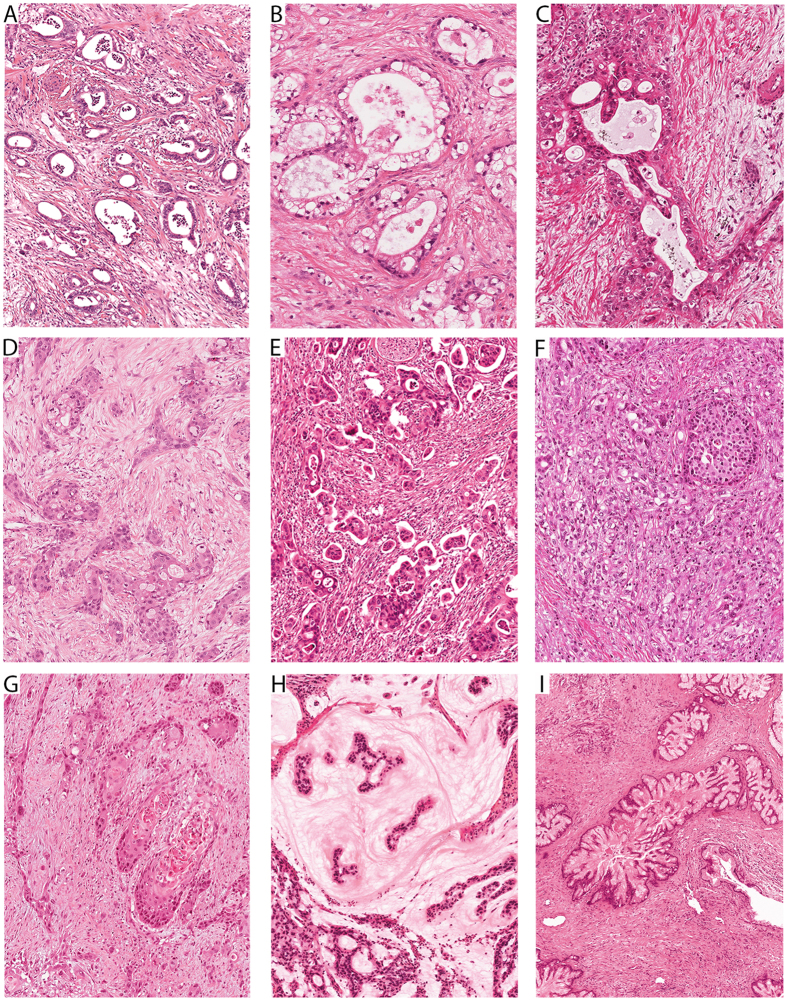
Spectrum of histologic patterns in pancreatic ductal adenocarcinomas (PDACs). (**A**) Conventional PDAC. (**B–F**) Combined PDACs with a dominant histological component: (**B**) Clear-cell component. (**C**) Cribriform component. (**D**) Gyriform component. (**E**) Micropapillary component. (**F**) Complex component. (**G**) Adenosquamous carcinoma. (**H**) Colloid carcinoma. (**I**) Papillary carcinoma.

**Figure 2:**
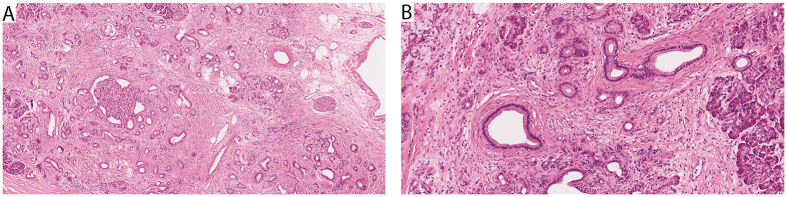
Tubular adenocarcinoma of the pancreas. (**A**) Low power view showing groups of well-differentiated infiltrating tubules surrounded by small cuffs of desmoplastic stroma. (**B**) Infiltrating well differentiated neoplastic glands closely imitating normal ducts.

The results are summarized in Table 2 and examples for each pattern are shown in Figs 1 and 2. The majority of the cases (92.1%, 163/177) were classified as either *conventional* PDACs (91/177, 51%), predominantly graded G2 (44%) or G3 (48%), or *combined* PDACs (72/177, 41%), predominantly graded G2 (37%) or G3 (61%). Two of the conventional PDACs contained at the periphery concomitant small (<1.5 cm) gastric type IPMNs and four had retention cysts. The *combined* PDACs showed as dominant histologic feature a conspicuous cribriform (17/177), clear-cell (16/177), papillary (17/177), gyriform (8/177), micropapillary (2/177) or complex (12/177) component (Fig. 1A–F). While the cribriform and clear cell combined PDACs were all G2 tumors, the gyriform, papillary, micropapillary and complex combined PDACs accounted for most (90%) of the G3 tumors. One PDAC with a clear cell component was associated with a pancreato-biliary type IPMN, and another PDAC with a complex component with a gastric type IPMN. A minority of tumors (7.9%, 14/177) fulfilled the criteria of PDAC *variants* and special pancreatic carcinomas. There were two G3-adenosquamous carcinomas (Fig. 1G), two G1-colloid carcinomas (Fig. 1H), six G2-papillary carcinomas (Fig. 1I), one G2-medullary carcinoma (not shown), and three G1-tubular carcinomas (Fig. 2). One of the colloid carcinomas was associated with an intestinal type IPMN. Two thirds of the papillary carcinomas (4/6) were associated with IPMNs, either of gastric (1/6), intestinal (1/6) or pancreato-biliary type (2/6). The three tubular adenocarcinomas were composed of well-differentiated open tubules that infiltrated the parenchyma diffusely and were accompanied by abundant cellular desmoplastic stroma (Fig. 2). The tubules had oval, rounded or angular shapes and were lined by a single layer of mostly cuboidal cells, with little nuclear pleomorphism, inconspicuous nucleoli and scanty mitotic figures.

No morphological differences were observed between patients who received neoadjuvant therapy and those without neoadjuvant treatment (data not shown).

### Molecular features

*KRAS, TP53, CDKN2A/p16* and *SMAD4* mutations were found in various frequencies and combinations in all but one tumor (case # 62; confirmed by NGS). Most PDACs carried multiple mutations (two: 47/177, 25.6%; three: 85/177, 48%; four: 36/177, 20.3%), while tumors with one mutation (8/177, 4.5%) were rare.

**Figure 3:**
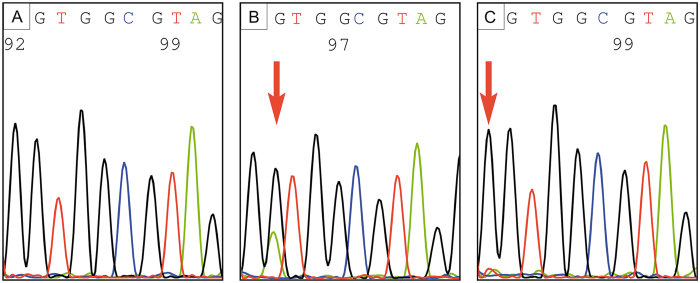
*KRAS* mutation analysis (direct sequencing) of codon 12 of exon 2. (**A**) Hotspot of exon 2 shows *KRAS* wildtype, (**B**) *KRAS* mutation (p.G12D, arrow), or (**C**) low-level mutation (p.G12C, arrow, faint red signal), as confirmed by next generation sequencing (NGS) (case #190).

*KRAS* was most frequently mutated (Table 3, Fig. 3), mainly in exon 2 (91%), and rarely in exon 3 (8.5%) and only once in both exons. No mutations were detected in exon 4. Almost all (98.8%, 162/164) *KRAS* mutations were detected by HRMA and Sanger sequencing (with expected detection limits of 10% and 15–20%, respectively (29)). Two low-level mutations (mutations with a low frequency in sequence reads) were identified by NGS (see also Fig. 3). All tumors with wildtype *KRAS* showed intact *BRAF* (codon 600).

**Figure 4:**
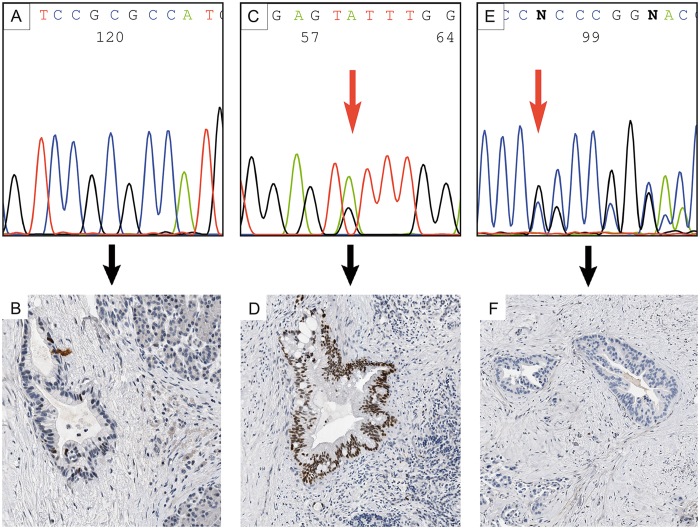
*TP53* analyses. **(A,B**) *TP53* wildtype is associated with nuclear TP53 expression in up to 24% of neoplastic cells. (**C,D**) *TP53* missense mutation (p.Y205C; exon 6) is associated with nuclear TP53 overexpression in ≥25% of neoplastic cells (mutation type 1). (**E,F**) *TP53* intragenic deletion or nonsense, frameshift or splicesite mutations (mutation type 2; here represented by an insertion-frameshift mutation p.P153*fs28; exon 5) is associated with loss of nuclear TP53 expression in neoplastic cells.

*TP53* wildtype and *TP53* mutations were associated with distinct p53 immunolabelling patterns depicted in Fig. 4. *TP53* Mutations were found in 139/177 tumors and detected in the known hotspots (exon 5–8), but not in exon 9. Mutation type 1 (defined as nuclear expression in ≥25% of tumor cells and missense mutation) was more common (52%, 92/177) than mutation type 2 (26.6%, 47/177; defined as absence of expression and presence of an intragenic deletion, a nonsense, a frameshift or splice site mutation) (Fig. 4).

**Figure 5:**
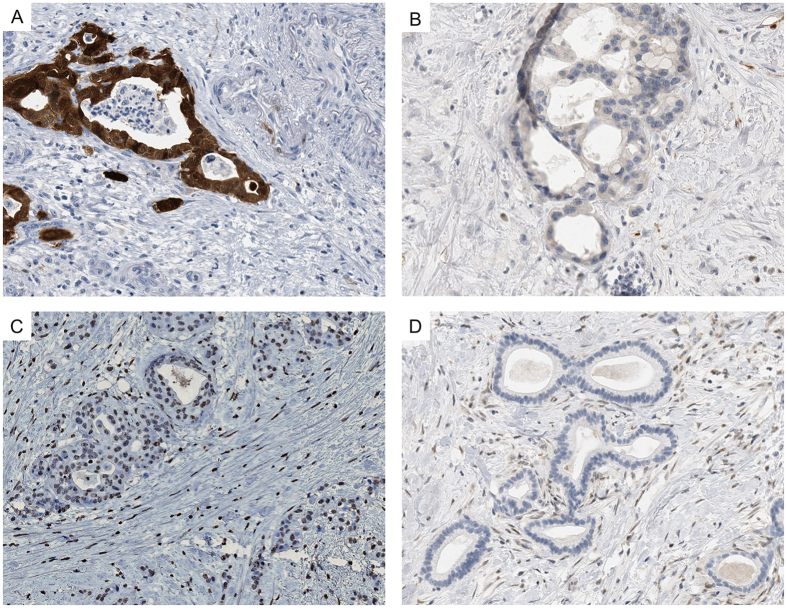
Immunohistochemical analysis of CDKN2A/p16 and SMAD4. (**A**) Strong nuclear and cytoplasmatic staining of CDKN2A/p16 in neoplastic cells indicating the presence of an intact gene. (**B**) No labeling of CDKN2A/p16 in neoplastic cells indicating either a deletion, inactivating mutation, or promoter hypermethylation. (**C**) Nuclear SMAD4 immunolabeling of neoplastic cells indicating the presence of an intact protein. (**D**) Loss of SMAD4 expression in >90% of neoplastic cells indicating a deletion or inactivating mutation of the gene.

*CDKN2A/p16* alterations, recorded in 75% of the tumors, were detected by loss of protein expression (130 tumors), which was confirmed by loss of heterozygosity (LOH) in 58 tumors. Three additional tumors showed intact expression despite presence of LOH. Loss of SMAD4 expression was recorded in one third of the cases (Table 3, Fig. 5).

Interestingly, altered *CDKN2A/p16* was significantly more common in patients with positive lymph nodes (p = 0.02). No significant differences between lymph node negative and positive patients were observed for number of mutations, *KRAS, TP53*, SMAD4 and tumor morphology.

Four investigated metastases (3 from the liver and one from the peritoneum) had the same *KRAS* mutations as the primary. In case # 5 a low-level mutation in the primary tumor (p.G12D, 3%) was associated with a high-level mutation (p.G12D) in the liver metastasis. In case # 9 intact *TP53* in the primary tumor was associated with a strong (>90%) nuclear labeling in the peritoneal metastasis.

No differences were observed in the mutational status of patients who received neoadjuvant therapy compared to the large group of patients without neoadjuvant therapy (data not shown).

### Correlation of molecular with morphologic features

*KRAS* wildtype was significantly more commonly detected in variants than classical PDACs (conventional or combined) (p = 0.035) (Table 4). In detail, the group of *KRAS* wildtype tumors (7.3%, 13/177) included *conventional* PDACs (7/13), *combined* PDACs (3/13), and one colloid, one medullary and one tubular carcinoma. The *KRAS* wildtype tubular carcinoma and one *KRAS*-mutated tubular carcinoma also lacked alterations in the three other genes (Table 5), a result that was confirmed by additional extended gene analysis. One of the two *KRAS*-mutated tubular carcinomas harbored *CDKN2A/p16* mutations (Table 5). All papillary carcinomas were associated with mutated *KRAS* and lacked mutated *GNAS*^18^, irrespective of the presence of an associated IPMN. *CDKN2A/p16* alterations were more strongly associated (p = 0.016) with *combined* PDACs (60/71) than with *conventional* PDACs (29/91) (Table 4).

### Clinical outcome correlated to morphologic and molecular features

At the time of survival analysis, 33 of 146 patients (22.6%) were alive. Median follow-up for all patients was 19.8 months (2.5–75.1 months) and median follow-up for patients alive was 49.4 months (25.9–75.1 months). Overall survival rates were 72% for one year, 29% for three years and 21% for five years.

Patients with colloid, medullary, tubular or papillary carcinoma survived significantly longer than patients with *conventiona*l and *combined* PDACs (p = 0.04) (Table 2). Longest survival was seen in two of the patients with a tubular carcinoma. They were still alive at the completion of the study, with a survival of >68.8 and >55.3 months, respectively. On the contrary, patients with adenosquamous carcinomas had an extremely poor outcome (4.1 and 10.0 months) (Table 2).

Detailed analysis of the large group of patients with conventional and combined PDACs revealed that patients with a conventional PDAC and those with a cribriform type *combined* PDAC showed the most favorable overall median survival (22.7 and 28.7 months), followed by *combined* PDACs with clear-cell and papillary components (17.6 and 13.9 months) (Table 2). *Combined* PDACs with gyriform and complex components were associated with poor survival (12.5 and 10 months).

**Figure 6:**
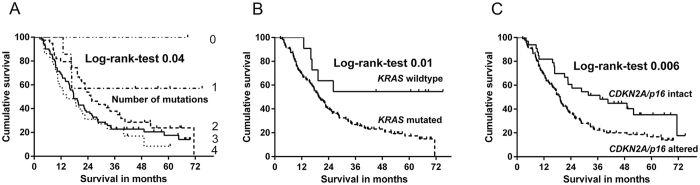
Kaplan-Meier survival curves in pancreatic adenocarcinomas correlated with molecular status. (**A**) Significant correlation of median patient survival with number of mutations. (**B**) Patients with *KRAS* wildtype have a significantly better overall survival than patients with mutated *KRAS*. (**C**) Patients with intact *CDKNA2/p16* have a significantly better overall survival than patients with altered *CDKNA2/p16*.

The median patient survival correlated significantly with the number of mutations (p = 0.04, Fig. 6A). Patients with no or low number of mutations (no mutation >68.6; one mutation >45; two mutations >25.3 months) survived longer than patients with tumors that harbored three (17.6 months) or four mutations (14.5 months).

Correlation of patient outcome and molecular features revealed a significant survival benefit in patients with wildtype *KRAS* (7.3%, 11/146) compared with that of *KRAS* mutated patients (92.7%, 135/146) (p = 0.018, median survival >45 *vs.* 19.7 months, Fig. 6B). Likewise, patients with intact *CDKN2A*/*p16* (22.8%, 33/145) lived significantly longer than those with altered *CDKN2A/p16* (77.2%, 112/145) (p = 0.006; 36.9 *vs*. 18.8 months, Fig. 6C). Significant prognostic results were further obtained by the combination of *CDKN2A*/p*16* and *KRAS*. All patients from the cohort followed for survival with an intact status of both genes (n = 4) were still alive at the end of the study. A median overall survival could therefore not be calculated. No prognostic significance was observed for mutated *TP53* (with no prognostic difference between *TP53* type 1 and type 2 mutations) and *SMAD4*.

Univariate survival analysis using log-rank test showed significant p-values for *KRAS, CDKN2A/p16*, number of mutations, pN and grading. In a backward selected multivariate Cox proportional Hazard model altered *KRAS* (HR 2.77, 95%-CI: 1.11–6.90; p = 0.019), pN1 (HR 2.66; 95%-CI: 1.67–4.26; p < 0.001) and grading G3 (HR 3.66, 95%-CI: 1.57–8.54; p = 0.001) were independent predictive variables for survival.

## Discussion

Reliable prognostic markers for pancreatic ductal adenocarcinoma (PDAC) patients are so far rare. By analyzing the expression profile of primary PDACs, and human and mouse PDAC cell lines, Collisson recently identified molecular subtypes of PDAC that differed in clinical outcome and drug response^11^. Other studies revealed an association between expression as well as mutational status of key tumor suppressor and oncogenes and patient survival^16^,^19^. In all these studies, the molecular profile of PDACs is not or only vaguely correlated to the individual morphology of the tumors.

Here we present data of a correlative study on histopathology, molecular profile, and survival in PDACs and related carcinomas of 177 resected patients, with the aim to find prognostic relevant features. As expected, the overall outcome of our patient cohort was bad. Nearly two thirds of patients survived less than 24 months and the 5-year survival did not exceed 21%. However, within this cohort there were patients who survived for up to four years and longer, and whose tumors had a special histopathology and/or molecular status.

Histopathological heterogeneity in PDACs has long been recognized, but it has not been defined in detail, -with the exception of the definition of histological grade and histological variants. Here we classified the pancreatic carcinomas according to a defined growth pattern into three groups. The first group included the *conventional* type PDACs, which showed an equal mixture of various histological elements (for details see Material and Methods). The second group encompassed the *combined* PDACs, which were characterized by a dominant histological component (defined as involving more than 30% of the tumor area). The third group contained PDAC *variants* (i.e. adenosquamous carcinoma, colloid carcinoma, papillary carcinoma) and special adenocarcinomas such as medullary carcinoma and tubular carcinoma. By analyzing the survival of patients who were ascribed to the various morphological types, we found and confirmed that the colloid carcinoma, the medullary carcinoma and the tubular carcinoma showed a better outcome than conventional PDACs and particularly adenosquamous carcinoma^20^,^21^,^22^,^23^. Patient with a *conventional* PDAC or a PDAC with dominant cribriform component survived longer than patients with *combined* PDAC and other histological components, such as the clear cell, the papillary, the gyriform and, in particular, the complex component.

PDACs typically harbor *KRAS* mutations, followed by mutations of *CDKN2A/p16, SMAD4* and *TP53*^10^. These genes are considered the driver genes of PDAC. In addition, there is a multitude of other, but much less frequent, gene alterations, as revealed by whole genome sequencing analysis^6^,^7^. In our patients, in whom the molecular results were obtained by Sanger methodology and completed in selected cases by NGS of a large PDAC gene panel including the 40 most commonly mutated genes, *KRAS* was found to be mutated in 92.7%*, TP53* in 78.5%, *CDKN2A/p16* in 72.9% and *SMAD4* in 37.3%. Most of these alterations coexisted in individual tumors and two third of the PDAC patients (68%) harbored alterations in three or all four genes.

**Figure 7:**
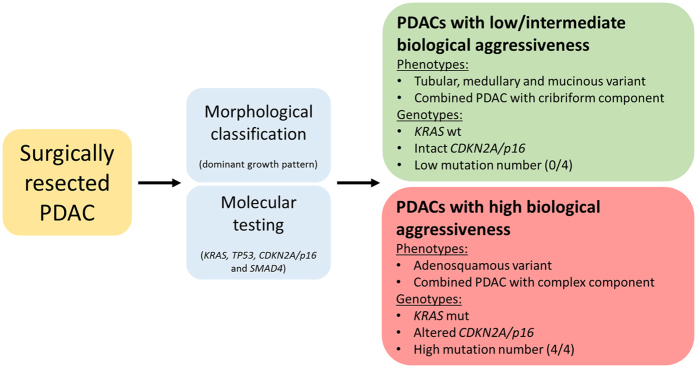
Risk stratification after surgical resection of PDAC. Proposed scheme to identify PDACs with low/intermediate and high biological aggressiveness based on morphological classification and molecular testing of key genes.

Correlation of the individual mutational status with the respective histopathology and survival data revealed a number of findings with prognostic significance. First, the number of mutations per tumor was of prognostic relevance. Patients with one, two or three mutations survived longer than those with alterations in all four genes (>45 *vs*. 25.3 *vs*. 17.6 *vs*. 14.5 months). Although these results confirm previous reports^16^, so far the correlation between gene status and phenotype has not been analysed in detail. Among the tumors with low number of mutations and prolonged survival, there are particularly colloid carcinomas, medullary carcinomas and tubular type carcinomas, while adenosquamous carcinomas, papillary carcinomas, and the *combined* PDAC with a complex pattern belong to the carcinomas with a high mutational frequency and poor survival (Fig. 7). Medullary and colloid carcinomas are known for their low prevalence of somatic mutations and good prognosis^20^,^21^,^22^,^24^. Conversely, adenosquamous carcinoma is well known for its many somatic mutations and aggressive behavior^25^,^26^. The papillary carcinoma variant seems in many cases to be the invasive component of an IPMN^27^, since IPMNs either of the pancreato-biliary (2/6), intestinal (1/6) or gastric type (1/6) were found in four of our six cases. However, it may also occur without an associated IPMN, since in two cases an associated IPMN was not found, and this was also true in another recently reported series of 10 papillary cystic PDACs^28^.

Second, patients with either wild type *KRAS* or *CDKN2A/p16* had a better outcome than those with mutations in these genes, while loss of SMAD4 and intact or altered *TP53* was of no prognostic relevance. The statistical significance of wildtype *KRAS* proved to be so strong that multivariate analysis identified the mutational status of *KRAS* as an independent prognostic marker. This result confirmed the recently reported survival benefit for PDAC patients with intact *KRAS*^12^,^13^,^14^,^15^. The largest of these studies by Sinn, included 153 PDAC patients and reported a significantly decreased median survival of 12.7 months for patients with mutated *KRAS* versus 20.7 months for patients with wild type *KRAS*^15^. In our study, the corresponding data were 19.7 months for mutated *KRAS versus* >45 months with wild type *KRAS*. The longer survival in our patients may be due to a better selection on the basis of a more extensive *KRAS* analysis including exon 2 to 4, in contrast to Sinn’s study that only focused on *KRAS* exon 2. This methodological difference probably also explains, why in Sinn’s study the *KRAS* mutation rate of 68% is much lower than in our study with a rate of 93%.

Among the tumors with a wildtype *KRAS* status were a medullary carcinoma, whose particular genetic and biologic features have been described^20^,^21^,^22^,^23^, and a tubular adenocarcinoma. This latter type of carcinoma, which corresponds by grade to a G1 tumor (WHO 2010), may be considered a conventional PDAC with a strictly well differentiated tubular differentiation. It shows great similarities regarding its morphology, low frequency, rare mutations and long survival to the tubular type carcinoma of the breast^29^,^30^,^31^,^32^. One of the three tubular adenocarcinomas that we identified, not only had an intact *KRAS* gene, but also harbored no alterations in the 40 most commonly altered PDAC genes, including *CDKN2A/p16, TP53* and *SMAD4*. Moreover, the patient with this tumor is still alive (see Table 4, case # 62), with a follow-up for >68.6 months. The other two patients with tubular carcinomas had *KRAS* and/or CDKN2A/p16 alterations, but no loss of *SMAD4* or *TP53* mutations (details see Table 4), and survived for >55.3 and 19.3 months.

The relevance of mutated *KRAS* as a prognosticator in PDAC, a feature shared with bile duct cancer^33^, is biologically most likely related to its driver function. Evidence that *KRAS*, in interaction with *TP53, CDKN2A/p16* and *SMAD4*, is a driver gene comes from genetically engineered *KRAS* mouse models of pancreatic cancer^10^,^34^,^35^,^36^,^37^,^38^,^39^,^40^,^41^. Moreover, recently developed mouse models in which mutated *KRAS* can be switched on and off, have impressively demonstrated that continuous oncogenic *KRAS* signaling is essential for both progression and maintenance of PDAC^42^,^43^ and its metastases^44^.

Patients with wildtype *CDKN2A/p16*, like patients with intact *KRAS,* lived significantly longer than those with altered genes (p = 0.006; 36.9 months *vs*. 18.8 months). Though multivariate analysis failed to identify this gene constellation as an independent prognostic factor, *CDKN2A/p16* seems to play a role in patient outcome. All our patients without alterations in *CDKN2A/p16* and *KRAS* were still alive at the completion of the study. On the other hand, loss of *CDKN2A/p16,* as was recently reported, seems to be associated with lymphatic invasion and widespread metastasis^19^ and was significantly associated with lymphatic spread in our study. Interestingly, subgroup analysis revealed a significant higher *CDKN2A/p16* mutation rate in *combined* PDACs with a dominant histological component compared to *conventional* PDACs. This suggests that a driver gene might be linked to the presence of dominant histological components in PDACs.

The prognostic relevance of *SMAD4* has been the subject of controversial discussions over the last years. While several studies associated the loss of *SMAD4* with poor prognosis or early metastatic disease^14^,^19^,^45^,^46^, others were unable to confirm these results^16^,^47^,^48^. Likewise, no differences in the survival data in patients with intact and loss of SMAD4 were observed in our study (p = 0.15, median overall survival 22.2 vs. 17.6 months).

In summary, our findings specify the prognostic relationship between the histopathology and molecular profile, based on the morphologic stratification of PDACs in subtypes and variants, and the mutational status of the four driver genes, *KRAS, CDKN2A/p16, SMAD4* and *TP53*. Because our data suggest that PDAC subgroups can be identified (low to intermediate aggressive PDACs versus highly aggressive PDACs, see Fig. 7), tailored therapy options may be discussed. Patients with altered *CDKN2A/p16* might benefit from more aggressive preoperative therapies, whereas patients with wildtype *KRAS* might best be treated by upfront surgery followed by adjuvant therapies. In view of these potential therapies it should be considered to include the status of the four driver genes into the pathological reporting of PDACs in the future^12^,^13^,^14^,^15^,^49^. Given the potential clinical implications of our results, validation in independent PDAC cohorts is of utmost importance.

## Material and Methods

The study was approved by the ethics committee of the TU München, Germany (documents no. 1926/2007 and 126/2016S). Written informed consent was obtained from all patients. All methods were performed in accordance with the relevant guidelines and regulations.

### Patients

From 07/2007 to 07/2011 200 patients underwent an elective pancreatic resection at the Department of Surgery, Klinikum rechts der Isar, TU München, Germany, with a final histopathologic diagnosis of PDAC. Associated and concomitant intraductal papillary mucinous neoplasms (IPMN)^50^ were reported. IPMNs with only minimally invasive component were excluded. TNM and grading followed the WHO recommendations^17^. Patients with a family history of PDAC were excluded from the study.

### Clinical data and follow up

Clinical, demographic and macroscopic information was obtained from a patient database and by reviewing the medical charts and pathology reports. Follow up on the patients’ conditions was obtained by clinical record, directly contacting the patients and/or their physicians. Patients with neoadjuvant therapy (n = 14, details see Supplementary Table 1), distant metastasis/UICC stage IV disease (n = 11, thereof six liver metastases, four peritoneal metastases and one pulmonary metastasis, details see Supplementary Table 2) and/or arterial resection (n = 4), perioperative death/death due to complications (n = 1) and recurrence surgery (n = 1) were excluded from survival analysis. Three patients were lost to follow-up.

### Histologic analysis

All PDACs were histologically classified into *conventional* PDACs, *combined* PDACs in which, in addition to the classical tubular growth pattern, a special histologic component was present in more than 30% of the tumor area, and *variants* with a special pattern in at least 50% of the tumor area. PDACs with a conventional morphology were largely composed of well- to moderately developed tubular and duct-like structures and showed only few other structures, such as glands with clear cell morphology, cribriform architecture, papillary epithelial lining and individual pleomorphic cells^17^. Combined PDACs with dominant histological features showed either a clear-cell, cribriform, gyriform, papillary, micropapillary or complex component against a background of tubular architecture (see Fig. 1). The complex component was characterized by small irregular glands mixed with solid or cribriform cell sheets and individual pleomorphic cells. PDAC variants included colloid, adenosquamous, and papillary carcinoma (see Fig. 1A–F). Among the pancreatic carcinomas that have not yet been regarded as PDAC variants are medullary and tubular carcinoma. The tubular adenocarcinoma is separated from conventional PDAC by its entirely well differentiated tubular architecture that is characterized by small tubular glands diffusely infiltrating the pancreatic parenchyma and difficult to distinguish from equally sized normal ducts (see Fig. 2).

### Immunohistochemical analysis

All stainings were run on an automated immunostainer with an iVIEW DAB detection kit (Ventana Medical Systems, Roche, Mannheim, Germany) according to the company’s protocols for open procedures with slight modifications, for details see Supplementary materials and methods.

### Molecular analysis

Molecular analysis was performed on extracted DNA from manually microdissected FFPE tumor tissue, for details see Supplementary materials and methods and Supplementary Table 1.

### Statistical analyses

The statistical analyses performed are described in Supplementary materials and methods.

## Additional Information

**How to cite this article:** Schlitter, A. M. *et al*. Molecular, morphological and survival analysis of 177 resected pancreatic ductal adenocarcinomas (PDACs): Identification of prognostic subtypes. *Sci. Rep.* **7**, 41064; doi: 10.1038/srep41064 (2017).

**Publisher's note:** Springer Nature remains neutral with regard to jurisdictional claims in published maps and institutional affiliations.

## Supplementary Material

Supplementary Information

## Figures and Tables

**Table 1:** Clinicopathological features of 177 patients with resected pancreatic adenocarcinomas.

	N, 177	%
Sex		
Male	96	54.2%
Female	81	45.8%
Age median in years	68 (31–88)	
**Tumor characteristics**		
pT		
1	4	2.3%
2	11	6.2%
3	142	80.2%
4	20	11.3%
pN		
0	52	29.4%
1	125	70.6%
Grading		
1	13	7.3%
2	72	40.7%
3	92	52.0%
Resection margin		
0	90	50.8%
1	61	34.5%
2	2	1.1%
X	24	13.6%

**Table 2:** Histological types of pancreatic ductal adenocarcinoma: frequency and survival.

Tumor type	Frequency	%	Type of associated IPMN	Median survival (months)
**Conventional ductal adenocarcinoma**	**91**	**51.4**	2 gastric	22.7
**Combined ductal adenocarcinoma**				
with cribriform component	17	9.6		28.7
with papillary component	17	9.6		13.9
with clear-cell component	16	9.0	1 pancreato-biliary	17.6
with complex component	12	6.7	1 gastric	10.7
with gyriform component	8	4.5		12.5
with micropapillary component	2	1.1		16.1
**Variants and special carcinomas**				
Adenosquamous carcinoma*	2	1.1		4.1
Colloidal/mucinous carcinoma*	2	1.1	1 intestinal	>64.3**
Medullary carcinoma*	1	0.5		>75.1**
Tubular carcinoma	3	1.7		>55.3**
Papillary carcinoma	6	3.4	2 pancreato-biliary, 1 intestinal, 1 gastric	20.6
**All tumors**	177	100		

**Table 3:** Molecular characteristics of pancreatic adenocarcinomas.

		N, 177	%
*KRAS*	Wildtype	13	7.3
	Mutated	164	93
*TP53*	Intact	38	21.5
	Altered	139	78.5
*CDKN2A/p16*	Intact	43	24
	Altered	133	75
	n.a.	1	0.6
SMAD4	Intact	112	63
	Altered	65	37

**Table 4:** Comparison between morphological phenotype, genotype, grading and survival.

Morphological phenotype	Conventional ductal adenocarcinoma (without components) (A1)	Combined ductal adenocarcinoma (with additional components) (A2)	Variants (B)
**N**	91	72	14
***KRAS***			
** Wildtype**	7	3	3
** Mutated**	84	69	11
** **p-value A1 *vs* A2^1^	0.35		
** **p-value A *vs* B^1^	0.035		
***CDKN2A/p16***			
** Intact**	29	11	3
** Altered**	62	60	11
** **p-value A1 *vs* A2^1^	0.016		
** **p-value A *vs* B^1^	0.78		
***TP53***			
** Intact**	22	11	5
** Altered**	69	61	9
** **p-value A1 *vs* A2^1^	0.16		
** **p-value A *vs* B^1^	0.17		
**SMAD4**			
** Intact**	57	45	10
** Altered**	34	27	4
** **p-value A1 *vs* A2^1^	0.98		
** **p-value A *vs* B^1^	0.51		
**Number of mutations**			
** 0**	0	0	1
** 1**	5	1	2
** 2**	30	14	3
** 3**	40	40	5
** 4**	16	17	3
** **p-value A1 *vs* A2^1^	0.09		
** **p-value A *vs* B^1^	0.004		
**Pathologic stage**			
**Primary tumour**			
** T1**	2	2	0
** T2**	3	8	0
** T3**	74	55	14
** T4**	12	7	0
** **p-value A1 *vs* A2^1^	0.23		
** **p-value A *vs* B^1^	0.55		
**Nodal status**			
** N0**	24	22	7
** N1**	67	50	7
** **p-value A1 *vs* A2^1^	0.60		
** **p-value A *vs* B^1^	0.12		
**Grading**			
** G1**	7	1	5
** G2**	4	27	6
** G3**	44	44	3
** **p-value A1 *vs* A2^1^	0.08		
** **p-value A *vs* B^1^	<0.001		
**Survival**			
** **(months)	22.7	15.5	34.1
** **p-value A1 *vs* A2^2^	0.07		
** **p-value A *vs* B^2^	0.06		

^1^ Chi^2^-test/Fisher’s exact test, ^2^Log-rank-test.

**Table 5:** Clinicopathologic and molecular features of pancreatic tubular adenocarcinoma.

ID	Sex	Age	Survival (months)	Site/Size (cm)	Grading	pT	pN	cM	*KRAS*	*TP53*	*CDKN2A/p16*	SMAD4
# 44	F	82.6	19.3	head/4.2	G1	T3	N1	M0	mut (p.G12V)	intact	altered	intact
# 62	M	74.9	>68.6, still alive	head/3.8	G1	T3	N0	M0	wt	intact	intact	intact
# 190	M	50.0	>55.3, still alive	head/3.5	G1	T3	N1	M0	mut (p.G12C low-level mutation, 6%)	intact	intact	intact

## References

[b1] Spath C. . Strategies to improve the outcome in locally advanced pancreatic cancer. Minerva Chir 70, 97–106 (2015). 25658301

[b2] Siegel R. L., Miller K. D. & Jemal A. Cancer statistics, 2015. CA Cancer J Clin 65, 5–29, doi: 10.3322/caac.21254 (2015). 25559415

[b3] Esposito I., Konukiewitz B., Schlitter A. M. & Kloppel G. Pathology of pancreatic ductal adenocarcinoma: facts, challenges and future developments. World J Gastroenterol 20, 13833–13841, doi: 10.3748/wjg.v20.i38.13833 (2014). 25320520 PMC4194566

[b4] Esposito I., Segler A., Steiger K. & Kloppel G. Pathology, genetics and precursors of human and experimental pancreatic neoplasms: An update. Pancreatology, doi: 10.1016/j.pan.2015.08.007 (2015). 26365060

[b5] Wood L. D. & Hruban R. H. Pathology and molecular genetics of pancreatic neoplasms. Cancer J 18, 492–501, doi: 10.1097/PPO.0b013e31827459b6 (2012). 23187835 PMC4013751

[b6] Witkiewicz A. K. . Whole-exome sequencing of pancreatic cancer defines genetic diversity and therapeutic targets. Nat Commun 6, 6744, doi: 10.1038/ncomms7744 (2015). 25855536 PMC4403382

[b7] Waddell N. . Whole genomes redefine the mutational landscape of pancreatic cancer. Nature 518, 495–501, doi: 10.1038/nature14169 (2015). 25719666 PMC4523082

[b8] Rozenblum E. . Tumor-suppressive pathways in pancreatic carcinoma. Cancer Res 57, 1731–1734 (1997). 9135016

[b9] Venter J. C. . The sequence of the human genome. Science 291, 1304–1351, doi: 10.1126/science.1058040 (2001). 11181995

[b10] Jones S. . Core signaling pathways in human pancreatic cancers revealed by global genomic analyses. Science 321, 1801–1806, doi: 10.1126/science.1164368 (2008). 18772397 PMC2848990

[b11] Collisson E. A. . Subtypes of pancreatic ductal adenocarcinoma and their differing responses to therapy. Nat Med 17, 500–503, doi: 10.1038/nm.2344 (2011). 21460848 PMC3755490

[b12] Lee J. . Impact of epidermal growth factor receptor (EGFR) kinase mutations, EGFR gene amplifications, and KRAS mutations on survival of pancreatic adenocarcinoma. Cancer 109, 1561–1569, doi: 10.1002/cncr.22559 (2007). 17354229

[b13] Ogura T. . Prognostic value of K-ras mutation status and subtypes in endoscopic ultrasound-guided fine-needle aspiration specimens from patients with unresectable pancreatic cancer. J Gastroenterol 48, 640–646, doi: 10.1007/s00535-012-0664-2 (2013). 22983505

[b14] Shin S. H. . Genetic alterations of K-ras, p53, c-erbB-2, and DPC4 in pancreatic ductal adenocarcinoma and their correlation with patient survival. Pancreas 42, 216–222, doi: 10.1097/MPA.0b013e31825b6ab0 (2013). 23344532

[b15] Sinn B. V. . KRAS mutations in codon 12 or 13 are associated with worse prognosis in pancreatic ductal adenocarcinoma. Pancreas 43, 578–583, doi: 10.1097/MPA.0000000000000077 (2014). 24681874

[b16] Yachida S. . Clinical significance of the genetic landscape of pancreatic cancer and implications for identification of potential long-term survivors. Clin Cancer Res 18, 6339–6347, doi: 10.1158/1078-0432.CCR-12-1215 (2012). 22991414 PMC3500447

[b17] Hruban R. H. . In WHO Classification of Tumors of the Digestive System (eds F.T. Bosman, F. Carneiro, R.H. Hruban & N.D. Theise) 292–299 (IARC, 2010).

[b18] Dal Molin M. . Clinicopathological correlates of activating GNAS mutations in intraductal papillary mucinous neoplasm (IPMN) of the pancreas. Ann Surg Oncol 20, 3802–3808, doi: 10.1245/s10434-013-3096-1 (2013). 23846778 PMC3842009

[b19] Oshima M. . Immunohistochemically detected expression of 3 major genes (CDKN2A/p16, TP53, and SMAD4/DPC4) strongly predicts survival in patients with resectable pancreatic cancer. Ann Surg 258, 336–346, doi: 10.1097/SLA.0b013e3182827a65 (2013). 23470568

[b20] Calhoun E. S. . BRAF and FBXW7 (CDC4, FBW7, AGO, SEL10) mutations in distinct subsets of pancreatic cancer: potential therapeutic targets. Am J Pathol 163, 1255–1260, doi: 10.1016/S0002-9440(10)63485-2 (2003). 14507635 PMC1868306

[b21] Goggins M. . Pancreatic adenocarcinomas with DNA replication errors (RER+) are associated with wild-type K-ras and characteristic histopathology. Poor differentiation, a syncytial growth pattern, and pushing borders suggest RER+. Am J Pathol 152, 1501–1507 (1998). 9626054 PMC1858440

[b22] Wilentz R. E. . Genetic, immunohistochemical, and clinical features of medullary carcinoma of the pancreas: A newly described and characterized entity. Am J Pathol 156, 1641–1651, doi: 10.1016/S0002-9440(10)65035-3 (2000). 10793075 PMC1876921

[b23] Wu J. . Whole-exome sequencing of neoplastic cysts of the pancreas reveals recurrent mutations in components of ubiquitin-dependent pathways. Proc Natl Acad Sci USA 108, 21188–21193, doi: 10.1073/pnas.1118046108 (2011). 22158988 PMC3248495

[b24] Adsay N. V. . Colloid (mucinous noncystic) carcinoma of the pancreas. Am J Surg Pathol 25, 26–42 (2001). 11145249 10.1097/00000478-200101000-00003

[b25] Borazanci E. . Adenosquamous carcinoma of the pancreas: Molecular characterization of 23 patients along with a literature review. World J Gastrointest Oncol 7, 132–140, doi: 10.4251/wjgo.v7.i9.132 (2015). 26380056 PMC4569590

[b26] Trikudanathan G. & Dasanu C. A. Adenosquamous carcinoma of the pancreas: a distinct clinicopathologic entity. South Med J 103, 903–910, doi: 10.1097/SMJ.0b013e3181ebadbd (2010). 20697320

[b27] Kloppel G., Basturk O., Schlitter A. M., Konukiewitz B. & Esposito I. Intraductal neoplasms of the pancreas. Semin Diagn Pathol 31, 452–466, doi: 10.1053/j.semdp.2014.08.005 (2014). 25282472

[b28] Kelly P. J. . Cystic papillary pattern in pancreatic ductal adenocarcinoma: a heretofore undescribed morphologic pattern that mimics intraductal papillary mucinous carcinoma. Am J Surg Pathol 36, 696–701, doi: 10.1097/PAS.0b013e318249ce1c (2012). 22367300

[b29] Rakha E. A., Pinder S. E., Shi S. J. & Tsuda H. In WHO Classiciation of Tumours of the Breast Vol. 4 (eds S.R. Lankhani .) (IARC, 2012).

[b30] Rakha E. A. . Tubular carcinoma of the breast: further evidence to support its excellent prognosis. J Clin Oncol 28, 99–104, doi: 10.1200/JCO.2009.23.5051 (2010). 19917872

[b31] Riener M. O. . Microarray comparative genomic hybridization analysis of tubular breast carcinoma shows recurrent loss of the CDH13 locus on 16q. Hum Pathol 39, 1621–1629, doi: 10.1016/j.humpath.2008.02.021 (2008). 18656243

[b32] Diab S. G. . Tumor characteristics and clinical outcome of tubular and mucinous breast carcinomas. J Clin Oncol 17, 1442–1448 (1999). 10334529 10.1200/JCO.1999.17.5.1442

[b33] Churi C. R. . Mutation profiling in cholangiocarcinoma: prognostic and therapeutic implications. PLoS One 9, e115383, doi: 10.1371/journal.pone.0115383 (2014). 25536104 PMC4275227

[b34] Biankin A. V. . Pancreatic cancer genomes reveal aberrations in axon guidance pathway genes. Nature 491, 399–405, doi: 10.1038/nature11547 (2012). 23103869 PMC3530898

[b35] Di Micco R. . Oncogene-induced senescence is a DNA damage response triggered by DNA hyper-replication. Nature 444, 638–642, doi: 10.1038/nature05327 (2006). 17136094

[b36] Guerra C. . Chronic pancreatitis is essential for induction of pancreatic ductal adenocarcinoma by K-Ras oncogenes in adult mice. Cancer Cell 11, 291–302, doi: 10.1016/j.ccr.2007.01.012 (2007). 17349585

[b37] Hingorani S. R. . Preinvasive and invasive ductal pancreatic cancer and its early detection in the mouse. Cancer Cell 4, 437–450 (2003). 14706336 10.1016/s1535-6108(03)00309-x

[b38] Mazur P. K. & Siveke J. T. Genetically engineered mouse models of pancreatic cancer: unravelling tumour biology and progressing translational oncology. Gut 61, 1488–1500, doi: 10.1136/gutjnl-2011-300756 (2012). 21873467

[b39] Morris J. P. t., Wang S. C. & Hebrok M. KRAS, Hedgehog, Wnt and the twisted developmental biology of pancreatic ductal adenocarcinoma. Nat Rev Cancer 10, 683–695, doi: 10.1038/nrc2899 (2010). 20814421 PMC4085546

[b40] Pylayeva-Gupta Y., Grabocka E. & Bar-Sagi D. RAS oncogenes: weaving a tumorigenic web. Nat Rev Cancer 11, 761–774, doi: 10.1038/nrc3106 (2011). 21993244 PMC3632399

[b41] Seidler B. . A Cre-loxP-based mouse model for conditional somatic gene expression and knockdown *in vivo* by using avian retroviral vectors. Proc Natl Acad Sci USA 105, 10137–10142, doi: 10.1073/pnas.0800487105 (2008). 18621715 PMC2481330

[b42] Collins M. A. . Oncogenic Kras is required for both the initiation and maintenance of pancreatic cancer in mice. J Clin Invest 122, 639–653, doi: 10.1172/JCI59227 (2012). 22232209 PMC3266788

[b43] Ying H. . Oncogenic Kras maintains pancreatic tumors through regulation of anabolic glucose metabolism. Cell 149, 656–670, doi: 10.1016/j.cell.2012.01.058 (2012). 22541435 PMC3472002

[b44] Collins M. A. . Metastatic pancreatic cancer is dependent on oncogenic Kras in mice. PLoS One 7, e49707, doi: 10.1371/journal.pone.0049707 (2012). 23226501 PMC3513322

[b45] Boone B. A. . Loss of SMAD4 staining in pre-operative cell blocks is associated with distant metastases following pancreaticoduodenectomy with venous resection for pancreatic cancer. J Surg Oncol 110, 171–175, doi: 10.1002/jso.23606 (2014). 24665063

[b46] Singh P., Srinivasan R. & Wig J. D. SMAD4 genetic alterations predict a worse prognosis in patients with pancreatic ductal adenocarcinoma. Pancreas 41, 541–546, doi: 10.1097/MPA.0b013e318247d6af (2012). 22504380

[b47] Biankin A. V. . DPC4/Smad4 expression and outcome in pancreatic ductal adenocarcinoma. J Clin Oncol 20, 4531–4542 (2002). 12454109 10.1200/JCO.2002.12.063

[b48] Winter J. M. . Failure patterns in resected pancreas adenocarcinoma: lack of predicted benefit to SMAD4 expression. Ann Surg 258, 331–335, doi: 10.1097/SLA.0b013e31827fe9ce (2013). 23360922 PMC4431700

[b49] Singh A. . A gene expression signature associated with “K-Ras addiction” reveals regulators of EMT and tumor cell survival. Cancer Cell 15, 489–500, doi: 10.1016/j.ccr.2009.03.022 (2009). 19477428 PMC2743093

[b50] Basturk O. . A Revised Classification System and Recommendations From the Baltimore Consensus Meeting for Neoplastic Precursor Lesions in the Pancreas. Am J Surg Pathol 39, 1730–1741, doi: 10.1097/PAS.0000000000000533 (2015). 26559377 PMC4646710

