# Supplementary material for: Molecular, morphological and survival analysis of 177 resected pancreatic ductal adenocarcinomas (PDACs): Identification of prognostic subtypes
Source: Sci Rep. 2017 Feb 1;7:41064. doi: 10.1038/srep41064 (PMC5286512; doi:10.1038/srep41064)
Supplement: Supplementary Information [file srep41064-s1.pdf]

Supplementary information

Molecular, morphological and survival analysis of 177 resected pancreatic ductal adenocarcinomas (PDACs): Identification of prognostic subtypes.

Anna Melissa Schlitter[*,1], Angela Segler[*,1], Katja Steiger[1], Christoph W. Michalski[2], Carsten Jäger[3], Björn Konukiewitz[1], Nicole Pfarr[1], Volker Endris[4], Markus Bettstetter[5], Bo Kong[3], Ivonne Regel[6], Jörg Kleeff[7,8], Günter Klöppel[$,1] and Irene Esposito[$,6]

* both authors contributed equally; [§] shared senior authorship

[1] Institute of Pathology, Technische Universität München, Munich, Germany.
[2] Department of Surgery, University Hospital Heidelberg, Germany.
[3] Department of Surgery, Klinikum rechts der Isar, Technische Universität München, Munich, Germany.
[4] Institute of Pathology, University Hospital of Heidelberg, Heidelberg, Germany
[5] Molecular Pathology South-Bavaria, Munich, Germany.
[6] Institute of Pathology, Heinrich-Heine-University, Düsseldorf, Germany.
[7] The Royal Liverpool and Broadgreen University Hospitals, Prescot Street, Liverpool L7 8XP, United Kingdom.
[8] Department of General-, Visceral- and Pediatric Surgery, University Hospital Düsseldorf, Heinrich Heine University Düsseldorf, Germany.

Supplementary materials and methods

Histological and immunohistochemical analysis

The slides from the paraffin-embedded formalin-fixed tissues of all surgical resection specimens were reviewed by five pathologists (AMS, AS, BK, GK and IE). In 177 patients, sufficient amount of tumor tissue was available and a representative tissue block was selected in each case for further analyses. The tissue blocks were cut into 3-5 μm-thick sections and stained with hematoxylin and eosin. Histological and immunohistochemical analyses and DNA extraction were performed on representative tissue blocks and tissue microarrays with two to three 2-mm tissue cores/sample.

Immunohistochemical stainings were performed using antibodies against CDKN2A/p16 (clone E6H4, CINtec®, MTM Laboratories AG, Heidelberg, Germany; ready to use), TP53 (clone DO-7, DakoCytomation, Glostrup, Denmark; 1:200) and SMAD4 protein (clone B8, Santa Cruz Biotechnology, Santa Cruz, CA, USA; 1:25). All stainings were run on an automated immunostainer with an iVIEW DAB detection kit (Ventana Medical Systems, Roche, Mannheim, Germany) according to the company's protocols for open procedures with slight modifications. CDKN2A/p16 expression was evaluated on tissue microarrays. Loss of expression (indicating a deletion, inactivating mutation, or promoter hypermethylation) was recorded, if the neoplastic cells showed no nuclear or cytoplasmatic staining [1,2]. Nuclear TP53 immunolabeling was considered overexpressed, when ≥ 25% of the neoplastic cells were positive (type 1 mutation). Loss of TP53 expression was recorded, when all cells were negative, suggesting either an introgenic deletion, or a nonsense or frameshift mutation (type 2 mutation) [3-5]. Loss of SMAD4 expression (indicating a deletion or inactivating mutation of the gene) was recorded, if there was no cytoplasmic and nuclear staining in ≥90% of neoplastic cells [6]. Normal islets (CDKN2A/p16 and SMAD4) and normal acinar cells, lymphocytes and stromal cells (TP53 and SMAD4) served as positive internal controls.

Molecular analysis

Genomic DNA was extracted from primary tumors (n=177) and, if available distant metastases (n=4/11), using the FFPE tissue Kit (QIAGEN) for manual microdissection from paraffin embedded samples of representative tissue blocks. For molecular analyses of *KRAS* exons 2 and 3 a combination of real-time PCR with high-resolution melting analysis (HRMA) and Sanger sequencing was used as described before [7,8]. In cases without mutations in *KRAS* exons 2 and 3, an additional mutation analysis of exon 4 was performed by a combination of real-time PCR with high-resolution melting analysis using the following

primers: KRAS-4-I-F: taa tac gac tca cta tag gGT TAA GGA CTC TGA AGA TGT ACC TAT GG, KRAS-4-IR: GTC CTG AGC CTG TTT TGT GTC T, KRAS-4-II-F: taa tac gac tca cta tag gAC AGG CTC AGG ACT TAG CAA GA and KRAS-4-II-R: TTT CAG TGT TAC TTA CCT GTC TTG TCT. Likewise, *BRAF* analysis (codon 600) using HRMA was performed in all cases with intact KRAS. Molecular analyses of *TP53* (exons 5-9) in all included cases and of *GNAS* in selected cases (papillary carcinoma) were performed by Sanger sequencing; loss of heterozygosity analyses of *CDKN2A/p16* locus were performed with quantitative real-time PCR of different genomic regions, distal and proximal to *CDKN2A* and the *CDKN2A* locus itself, as described before [7,9]. In addition to Sanger sequencing, focused next generation panel sequencing was performed in three cases (# 5, 62 and 190). In detail, case # 5 showed a discrepancy between primary tumor and metastasis in Sanger sequencing of *KRAS*, case # 62 showed intact *KRAS, CDKN2A/p16, TP53* and SMAD4 and case # 190 revealed a faint signal in Sanger sequencing of *KRAS* (Figure 1C).

Library preparation and semiconductor sequencing

For next generation sequencing, DNA content was measured fluorometrically using the QuBit HS DNA Assay (Life Technologies). Determination of the DNA sequencing grade quality was done using a real-time qPCR-based method (RNAseP Detection system, Life Technologies). Amplicon library preparation was performed with the Ion AmpliSeq Library Kit v2.0. For mutation analysis, the pancreatic ductal adenocarcinoma/cholangiocarcinoma panel was employed (for details see supplementary table 1). The pancreatic ductal adenocarcinoma/cholangiocarcinoma panel consists of two primer pools yielding 285 amplicons that cover hotspot regions of 40 pancreatic ductal adenocarcinoma/cholangiocarcinoma-related genes (supplementary table 1; including *KRAS*, *p16/CDKN2A*, *TP53* and *SMAD4*). For amplification, approximately 10ng of DNA, determined by qPCR assay, were used. Briefly, the DNA was mixed with the primer pool and the AmpliSeq HiFi Master Mix in a 20µl reaction volume and transferred to a PCR cycler (Biometra, Göttingen, Germany). Upon completion of the PCR reaction, amplicons were partially digested using FuPa reagent, followed by the ligation of barcoded sequencing adapters (Ion Xpress Barcode Adapters, Thermo Fisher Scientific, Waltham, USA). The final library was purified using AMPure XP magnetic beads (Beckman Coulter, Krefeld, Germany) and quantified using qPCR (Ion Library Quantitation Kit, Thermo Fisher Scientific, Waltham, USA) on a StepOne*Plus* qPCR machine (Thermo Fisher Scientific, Waltham, USA). The individual libraries were diluted to a final concentration of 100pM. All libraries were pooled and processed to library amplification on Ion Spheres using Ion PI™ Template OT2 200 Kit v3. Unenriched libraries were quality-controlled using Ion Sphere quality control measurement on a QuBit instrument. After library enrichment (Ion OneTouch ES, Thermo

Fisher Scientific, Waltham, USA), the library was processed for sequencing using the Ion PI™ Sequencing 200 Kit v3 chemistry and the barcoded libraries were loaded onto a PI chip.

Data analysis

Raw sequencing data were processed using the implemented Torrent Suite Software (version 4.4.3) and aligned against the human genome (version hg19) using TMAP algorithm. For DNA mutation analysis, the aligned reads were processed using the build-in Variant Caller plugin (version 4.4.3). Variant annotation was performed using a custom build variant annotation pipeline in the CLC Genomics Workbench (version 8.0.2). For visualization of sequencing and fusion reads, the Integrative Genomics Viewer Browser (IGV, http://www.broadinstitute.org/igv/) was used. Variants were screened against the COSMIC (catalogue of somatic mutations in cancer) database [10] to identify already known somatic mutations and mutation types, respectively. The dbSNP database [11] was used to identify and exclude common germline variants from the analysis.

Statistical analyses

Continuous variables are reported as median (min.-max.). Categorical variables are summarized as frequency counts and percentages and were compared using Fischer's exact test or Pearsons's chi-square test, as appropriate. Overall survival was defined as time from resection until death or until last follow-up. Survival analysis was performed using the Kaplan-Meier method; differences were evaluated with the log-rank test. A multivariate Cox Proportional Hazard model was performed to estimate hazard ratios and prognostic factors on survival. A two-sided p value of <0.05 was considered as significant. All statistical analyses were performed using IBM SPSS, v22 for Windows (IBM Inc., USA).

Supplementary table 1. Treatment schedule of PDAC patients with neoadjuvant therapy

ID	Therapy	Duration/ Dose	Cycles	Survival (months)
**# 5**	Gemcitabine/Erlotinib	4 months	4	17.8
	Gemcitabine	2 months	2	
**# 15**	Radiotherapy	30 Gy		11.7
	5-Fluorouracil	1 month		
**# 19**	Gemcitabine/Cisplatin/Tarceva	9 months	9	77.7
	Oxaliplatin	4 months	4	
	Radiotherapy plus 5-Fluorouracil	36 Gy	3	
**# 26**	Gemcitabine	7 months	7	11.0
**# 36**	Epirubecin/Oxaliplatin/Xeloda	6 months	6	8.1
**# 37**	Gemcitabine/Oxaliplatin	5 months		13.0
	Gemcitabine/Oxaliplatin	2 months		
**# 48**	Radiotherapy	36 Gy		49.8
	5-Fluorouracil	1 month		
**# 61**	Gemcitabine/Oxaliplatin	6 months	12	5.9
**# 102**	Gemcitabine/Erlotinib	4 months	4	3.5
**# 128**	Gemcitabine/Erlotinib	2 months	2	13.6
**# 142**	Cisplatin	1 month		4.5
**# 152**	Gemcitabine/Oxaliplatin	3 months	3	4.1
**# 185**	Gemcitabine	4 months	4	24.5
**# 188**	Gemcitabine/ Oxaliplatin	6 months	10	8.4

Supplementary table 2. Metastases, perioperative

ID	Location	Survival (months)
**# 5**	Liver	17.8
**# 9**	Peritoneal	10.9
**# 21**	Liver	25.3
**# 30**	Peritoneal	13.4
**# 37**	Lung	13.0
**# 41**	Peritoneal	7.9
**# 43**	Liver	8.9
**# 53**	Liver	12.3
**# 70**	Liver	11.3
**# 73**	Liver	8.6
**# 75**	Peritoneal	8.1

References

1. Geradts J, Hruban RH, Schutte M*, et al.* Immunohistochemical p16INK4a analysis of archival tumors with deletion, hypermethylation, or mutation of the CDKN2/MTS1 gene. A comparison of four commercial antibodies. *Appl Immunohistochem Mol Morphol* 2000; **8**: 71-79.
2. Wilentz RE, Geradts J, Maynard R*, et al.* Inactivation of the p16 (INK4A) tumor-suppressor gene in pancreatic duct lesions: loss of intranuclear expression. *Cancer Res* 1998; **58**: 4740-4744.
3. Melhem MF, Law JC, el-Ashmawy L*, et al.* Assessment of sensitivity and specificity of immunohistochemical staining of p53 in lung and head and neck cancers. *Am J Pathol* 1995; **146**: 1170-1177.
4. Obata A, Eura M, Sasaki J*, et al.* Clinical significance of p53 functional loss in squamous cell carcinoma of the oropharynx. *Int J Cancer* 2000; **89**: 187-193.
5. Sjogren S, Inganas M, Norberg T*, et al.* The p53 gene in breast cancer: prognostic value of complementary DNA sequencing versus immunohistochemistry. *J Natl Cancer Inst* 1996; **88**: 173-182.
6. Wilentz RE, Su GH, Dai JL*, et al.* Immunohistochemical labeling for dpc4 mirrors genetic status in pancreatic adenocarcinomas : a new marker of DPC4 inactivation. *Am J Pathol* 2000; **156**: 37-43.
7. Schlitter AM, Jang KT, Kloppel G*, et al.* Intraductal tubulopapillary neoplasms of the bile ducts: clinicopathologic, immunohistochemical, and molecular analysis of 20 cases. *Mod Pathol* 2015; **28**: 1249-1264.
8. Schlitter AM, Born D, Bettstetter M*, et al.* Intraductal papillary neoplasms of the bile duct: stepwise progression to carcinoma involves common molecular pathways. *Mod Pathol* 2014; **27**: 73-86.
9. Ormanns S, Siveke JT, Heinemann V*, et al.* pERK, pAKT and p53 as tissue biomarkers in erlotinib-treated patients with advanced pancreatic cancer: a translational subgroup analysis from AIO-PK0104. *BMC Cancer* 2014; **14**: 624.
10. Forbes SA, Beare D, Gunasekaran P*, et al.* COSMIC: exploring the world's knowledge of somatic mutations in human cancer. *Nucleic Acids Res* 2015; **43**: D805-811.
11. Sherry ST, Ward MH, Kholodov M*, et al.* dbSNP: the NCBI database of genetic variation. *Nucleic Acids Res* 2001; **29**: 308-311.
